# Diagnoses, prevalence, and state-based federal spending for HIV prevention and treatment in the United States, 2006–2009

**DOI:** 10.1186/1742-6405-11-15

**Published:** 2014-06-06

**Authors:** Willie H Oglesby, Joseph L Smith, Sonia A Alemagno

**Affiliations:** 1 Department of Health Policy & Management, College of Public Health, Kent State University, PO Box 5190, Kent, OH 44242, USA; 2 Department of Health Policy & Management, College of Public Health, University of South Florida, 12901 Bruce B. Downs Blvd., Tampa, FL 33612, USA

**Keywords:** HIV, Resource allocation, Economic evaluation, HIV treatment, HIV prevention, United States

## Abstract

**Background:**

In response to an article published in 2012 by officials at the US Department of Health and Human Services (DHHS), an independent analysis of state-based federal resource allocation for HIV was conducted to determine if the funding accurately reflected diagnosis and prevalence rates.

**Methods:**

Total state-based federal funding for HIV, state-based funding for HIV prevention, and state-based funding for HIV treatment were compared to state-based HIV diagnosis and prevalence rates from 2006-2009.

**Results:**

Total state-based federal funding for HIV and funding for HIV prevention and treatment were highly correlated with HIV diagnosis and prevalence rates during the time horizon of the study; however, correlations between state-based HIV prevention funding and state-based HIV diagnosis rates were lower than the correlations between state-based HIV treatment funding and HIV prevalence.

**Conclusions:**

Our findings suggest that state-based federal resource allocation for HIV prevention and treatment may be better aligned with HIV diagnosis and prevalence rates than previously reported; however resource allocation for HIV prevention is less aligned than funding for HIV treatment signaling the need to reexamine state-based federal funding for HIV prevention.

## Background

Human immunodeficiency virus (HIV) is endemic in the United States, with an estimated 1.1 million people infected at the end of 2010 [1]. However, HIV infection is not evenly distributed across the country. From 2006 - 2009, HIV diagnosis and prevalence rates were 10 to 20 times higher in New York than North Dakota and are generally higher in Southeastern states and urban areas (see Additional file 1). To address the impact of HIV, the federal government provides funding to states to help reduce the spread of the disease and provide treatment services to those who are infected.

In 2012, officials (Mansergh and colleagues) at the US Department of Health and Human Services (DHHS) published an article [2] comparing total state-based federal funding for HIV to the number of living HIV and AIDS cases and HIV and AIDS prevalence rates. The results of their analysis indicated that funding was highly correlated with the total number of living AIDS cases (*r* = .88) and total number of living HIV cases (*r* = .84), but were less correlated with AIDS prevalence rates (*r* = .35) and HIV prevalence rates (*r* = .42). The researchers concluded that while federal funding for HIV overall is well correlated with the HIV and AIDS cases, more research is needed to better assess the alignment of federal resource allocation for HIV funding with the actual epidemiologic profile. This research responds to this call.

While the analysis conducted by Mansergh and colleagues [2] is an important first step in examining the alignment between federal resource allocation for HIV and the HIV burden, several methodological issues affect the interpretation and utility of the findings. Firstly, state-based HIV funding data from 2012 were correlated with epidemiologic data from 2008. While this time difference may reflect what was the most up-to-date information at the time of analysis and how actual funding decisions are made (delayed epidemiologic reporting at the time of grant submission and awarding), it does not reflect the association between the *actual* epidemiologic profile at the time the funding as provided to address it. Secondly, the analysis included US states and territories that did not have confidential name-based HIV infection reporting at the time the epidemiologic data were reported, which raises concerns about accuracy and reliability of the epidemiologic information from those states. Thirdly, only *prevalence* counts and rates were used in the analysis and not counts or rates of *new* diagnoses of HIV infection. The absence of *new* diagnosis reporting in the analysis limits the ability to assess the degree to which funding is directed to areas experiencing spikes in diagnoses. Lastly, the analysis was based on comparisons made at one point in time, which can be biased by anomalous events that occurred during that time period.

In light of these methodological limitations, a study was conducted to independently assess the association between state-based federal funding and HIV epidemiology using a different analytical framework that includes HIV diagnosis and prevalence rates (rather than HIV counts or AIDS data), state-based federal funding separated into funding for prevention and treatment (rather than just total federal funding), pair-wise comparisons at the same point in time (rather than comparisons across years), and comparisons over a time horizon of four years (rather than just one time period). The purpose of the study is to better understand the relationship between state-based federal spending for HIV prevention and treatment and HIV diagnosis and prevalence rates in the United States to help determine if the federal resource allocation for HIV reflects the epidemiologic profile in the states that receive funds.

## Findings

### Measures

State-based federal funding data for HIV were obtained through a special data request to State Health Facts, a project of the Henry J. Kaiser Family Foundation (KFF). The KFF maintains a large collection of data from a variety of sources, including total federal HIV grant funding to US states, territories, and the District of Columbia. The data originated from the National Alliance of State & Territorial AIDS Directors (NASTAD), which tracks this information over time and reports it to KFF. The data included state-based HIV funding from the Centers for Disease Control and Prevention (CDC), Housing Opportunities for Persons with AIDS (HOPWA), Substance Abuse and Mental Health Services Administration (SAMHSA), the U.S. Office of Minority Health (OMH), and the Ryan White HIV/AIDS Program.

In a sub-analysis, total state-based federal funding for HIV were separated into two categories based on the primary use of the funding. Funding for HIV *prevention* included funding primarily for direct-service programs designed to reduce the number of new HIV infections. Funding for HIV *treatment* included funding primarily for direct-service programs designed to increase treatment access, improve health outcomes, and address other health issues for People Living With HIV (PLWH). In order to determine the primary use of the funding, web pages, Requests for Proposals, award notices, and other information on the funder’s web sites were consulted. In several cases, state-based federal funding for HIV could not be easily categorized into prevention or treatment categories primarily because the funds could be used to support HIV activities in both areas. Only funds that were clearly designated as prevention or treatment were categorized as such in the sub-analysis (see Additional file 1 for categorization of funding in the sub-analysis).

HIV diagnosis and prevalence rates were obtained through a special data request to ATLAS, a project of the National Center for HIV/AIDS, Viral Hepatitis, STD, and TB Prevention (NCHHSTP). ATLAS was created by NCHHSTP to provide the public with interactive maps, graphs, tables, and figures showing geographic patterns and time trends of HIV and other diseases [3]. The data included state-level HIV diagnosis and prevalence rates for the 40 states with confidential name-based HIV infection reporting during the time horizon of the study (2006–2009). These states included AL, AK, AZ, AR, CO, CT, FL, GA, ID, IL, IN, IA, KS, KY, LA, ME, MI, MN, MS, MO, NE, NV, NH, NJ, NM, NY, NC, ND, OH, OK, PA, SC, SD, TN, TX, UT, VA, WV, WI, and WY. States without confidential name-based HIV infection reporting systems during the time horizon of the study were excluded from analysis due to concerns over the accuracy and reliability of information. In addition, AIDS prevalence and new AIDS case rates were not included in the analysis because the number of people living with an AIDS diagnosis are already included in the HIV rates and because the funding from programs used in the analysis, particularly treatment funding, benefit all PLWH, not just those with an AIDS diagnosis. Lastly, case counts were not used in the analysis, since case rates are a more widely used metric in the scientific literature and supported by the U.S. National HIV/AIDS Strategy [4].

### Analysis procedures

Descriptive statistics were performed to describe total state-based federal funding for HIV, state-based funding for HIV prevention, state-based funding for HIV treatment, HIV diagnosis rates, and HIV prevalence rates. Bivariate analyses using Pearson’s correlation and Spearman’s rank correlation were conducted to assess the association between funding for HIV and HIV diagnosis and prevalence rates. Pearson’s correlation was selected so that results could be compared to the Mansergh article [2]. However, tests of normality using Kolmogorov-Smirnov and Shapiro-Wilk revealed that the data were not normally distributed (significance set at p < .05), so Spearman’s rank correlations were also performed. All correlation coefficients in our analysis were significant at p < .01.

### Hypothesis

The hypotheses for the bivariate analyses were:

*H*_*0*_: There is no correlation between total state-based federal funding for HIV and HIV diagnosis rates.

*H*_*0*_: There is no correlation between total state-based federal funding for HIV and HIV prevalence rates.

*H*_*0*_: There is no correlation between total state-based federal funding for HIV prevention and HIV diagnosis rates.

*H*_*0*_: There is no correlation between total state-based federal funding for HIV prevention and HIV prevalence rates.

*H*_*0*_: There is no correlation between total state-based federal funding for HIV treatment and HIV diagnosis rates.

*H*_*0*_: There is no correlation between total state-based federal funding for HIV treatment and HIV prevalence rates.

## Results

The total amount of state-based federal funding for HIV included in the analysis was between $2.05 and $2.28 billion from 2006 to 2009. Of that, between $276 and $314 million were categorized as prevention and between $1.44 and $1.59 billion were categorized as treatment, which represent approximately 84% of the total state-based federal funding for HIV used in the analysis (see Additional file 1).

### Total state-based federal funding for HIV

**Figure 1:**
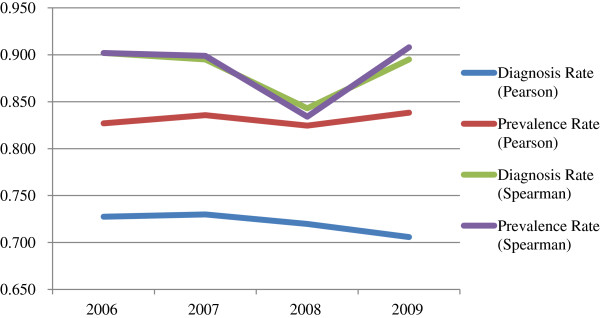
Correlations between total state-based federal funding for HIV and HIV diagnosis and prevalence rates by year.

As seen in Table 1, the Pearson’s and Spearman’s correlations between total state-based federal funding for HIV and HIV diagnosis rates ranged from .706 to .730 and .843 to .902, respectively. Pearson’s and Spearman’s correlations between total HIV funding and HIV prevalence rates were mostly higher and ranged from .825 to .838 and .834 to .908, respectively. As shown in Figure 1, Pearson’s correlations between total HIV funding and HIV prevalence rates remained fairly consistent from 2006 to 2009, but Pearson’s correlations with HIV diagnosis rates declined slightly over that time period. Spearman’s correlations between total HIV funding and HIV diagnosis and prevalence rates were higher than Pearson’s correlations. In addition, the Spearman’s correlations between total HIV funding and HIV diagnosis and prevalence rates were very similar to each other, with a sharp drop in 2008.

**Table 1:** **Correlations of state-based federal funding for HIV, HIV diagnosis rates, and HIV prevalence rates, 2006-2009**^**1**^

	**2006**				**2007**				**2008**				**2009**			
	**HIV diagnosis rate**		**HIV prevalence rate**		**HIV diagnosis rate**		**HIV prevalence rate**		**HIV diagnosis rate**		**HIV prevalence rate**		**HIV diagnosis rate**		**HIV prevalence rate**	
	***r***	***r***_***s***_	***r***	***r***_***s***_	***r***	***r***_***s***_	***r***	***r***_***s***_	***r***	***r***_***s***_	***r***	***r***_***s***_	***r***	***r***_***s***_	***r***	***r***_***s***_
Total state-based federal funding for HIV	.728	.902	.827	.902	.730	.895	.836	.899	.720	.843	.825	.834	.706	.895	.838	.908
State-based federal funding for HIV prevention	.700	.878	.826	.888	.698	.867	.835	.888	.696	.855	.832	.895	.664	.861	.823	.887
State-based federal funding for HIV treatment	.739	.915	.830	.911	.745	.901	.839	.902	.731	.817	.824	.797	.718	.908	.843	.915

^1^Data limited to the 40 states with confidential name-based HIV infection reporting during the time horizon of the study.

*r* = Pearson’s correlation coefficient.

*r*_*s*_ = Spearman’s rank correlation coefficient.

All correlations significant at p < .01.

### State-based funding for HIV prevention

**Figure 2:**
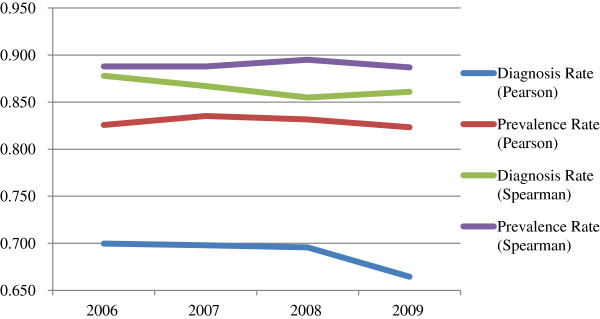
Correlations between total state-based federal funding for HIV prevention and HIV diagnosis and prevalence rates by year.

As seen in Table 1, the Pearson’s and Spearman’s correlations between total state-based federal funding for HIV prevention and HIV diagnosis rates ranged from .664 to .700 and .855 to .878, respectively. Pearson’s and Spearman’s correlations between HIV prevention funding and HIV prevalence rates were mostly higher and ranged from .823 to .835 and .887 to .895, respectively. As shown in Figure 2, Pearson’s correlations between HIV prevention funding and HIV prevalence rates remained fairly consistent from 2006 to 2009, but correlations with HIV diagnosis rates declined sharply 2008 to 2009. Spearman’s correlations between funding for HIV prevention and HIV diagnosis and prevalence rates were higher than Pearson’s correlations. However, Spearman’s correlations between funding for HIV prevention and HIV prevalence rates were consistently higher than the correlations between HIV prevention funding and diagnosis rates.

### State-based funding for HIV treatment

**Figure 3:**
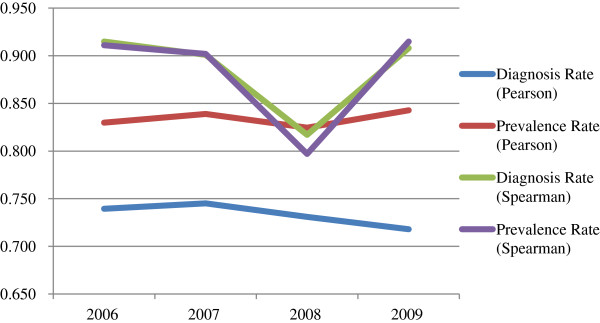
Correlations between total state-based federal funding for HIV treatment and HIV diagnosis and prevalence rates by year.

As seen in Table 1, the Pearson’s and Spearman’s correlations between total state-based federal funding for HIV treatment and HIV diagnosis rates ranged between .718 to .745 and .817 to .915, respectively. Pearson’s and Spearman’s correlations between HIV treatment funding and HIV prevalence rates were mostly higher and ranged between .824 to .843 and .797 to .915, respectively. As shown in Figure 3, Pearson’s correlations between HIV treatment funding and HIV prevalence rates remained fairly consistent from 2006 to 2009, but correlations with HIV diagnosis rates declined from 2007 to 2009. Spearman’s correlations between HIV treatment funding and HIV diagnosis and prevalence rates were very similar to each other, with a sharp drop in 2008.

## Discussion

The purpose of the study is to better understand the relationship between state-based federal spending for HIV prevention and treatment and HIV diagnosis and prevalence rates in the United States. To compare findings to those published by Mansergh and colleagues [2], Pearson’s correlations were performed; however, due to concerns over the normality of the data, Spearman’s correlations were also performed. In our analysis, Spearman’s correlations were higher than Pearson’s correlations in nearly all comparisons, which is likely due to the effect of outliers on the Pearson’s correlation calculation. The data were also not normally distributed in the Mansergh article, but only Pearson’s correlations were reported.

Overall, correlations between total state-based federal funding for HIV and HIV diagnosis and prevalence rates were high (*r* from .664 to .843 and *r*_*s*_ from .797 to .915), indicating that state-based federal funding for HIV is fairly well aligned with state-based HIV diagnosis and prevalence rates. These correlations are higher than those found by Mansergh and colleagues, who found only a modest correlation between HIV funding and HIV prevalence rates (*r* = .42). Several reasons may explain this difference.

Firstly, in the Mansergh article, states without confidential name-based HIV infection reporting were included in the analysis, which could have skewed their results. Secondly, the funding data gathered by Mansergh included Medicaid and Medicare spending, which our analysis did not. Thirdly, the HIV prevalence rates used by Mansergh were different than those used in our analysis. Although the average difference was small across all states in the analysis (~3%), in some states the HIV prevalence rates used by Mansergh were between -8% and 10% different than the rates we used. This is likely due to reporting corrections that were made after their analysis. Lastly, although the data were not normally distributed, Mansergh and colleagues only reported Pearson’s correlation coefficients, which makes their findings heavily influenced by outliers in the data.

In our sub-analysis, we found interesting differences in the correlations between funding for HIV prevention and treatment and HIV diagnosis and prevalence rates. For HIV treatment funding, a high correlation with HIV prevalence rates makes sense since the purpose of the funding is to provide services to people who are already HIV + and because the Ryan White HIV/AIDS Program includes HIV prevalence counts in its funding formulas. States with larger proportions of their population living with HIV need more resources than states with smaller proportions of PLWH. However, since funding for HIV *prevention* is primarily directed at reducing the number of new HIV diagnoses, higher correlations between HIV prevention funding and new HIV diagnosis rates were expected, even though effective treatment can also reduce HIV transmission [5].

### Limitations

This research also has its limitations. Firstly, it relied solely on data reported to and by the Kaiser Family Foundation and ATLAS, which may have been inaccurate or incomplete at the time the information was gathered. Secondly, the assignment of funding into “prevention” and “treatment” categories was conducted by the researchers and not the funders. While we made every effort to correctly assign the funding to the appropriate category, some may have been categorized incorrectly. Thirdly, this analysis is based only on the 40 states that had confidential name-based HIV infection reporting during the time horizon of the study. Although we limited the analysis to these states for reasons of accuracy and reliability, because of the restricted number of jurisdictions, these findings may not necessarily be representative of the association between HIV funding and HIV diagnosis and prevalence rates for all US states and territories. Fourthly, the federal funding used in our analysis did not include Medicaid and Medicare spending, which limits the findings to those non-medical services. Lastly, it should be noted that the correlations presented here are statistical associations and do not account for all of the various factors that would affect HIV transmission and treatment, including access to prevention programs and health care. It also does not account for undiagnosed HIV infections. Readers should consider these limitations when interpreting these findings.

## Conclusions

Overall, state-based federal resource allocation for HIV may be better aligned with the HIV burden than previously reported, as evidenced by high correlations between HIV funding and HIV diagnosis and prevalence rates. Correlations between HIV prevention funding and HIV diagnosis rates, however, are lower than correlations between HIV treatment funding and HIV prevalence rates, which indicate that state-based federal funding for HIV prevention may not be as well aligned as the state-based federal funding for HIV treatment. Additional research is needed to determine the optimal resource allocation model for federal HIV funding so that resources are directed to states that need it the most.

## Competing interests

The authors declare that they have no competing interests.

## Authors’ contributions

WHO designed the study, analyzed the data, and prepared the manuscript. JS collected and verified the data and provided critical revisions to the manuscript. SAA provided critical revisions to the manuscript. All authors read and approved the final manuscript.

## Supplementary Material

Additional file 1 HIV Diagnosis Rates, HIV Prevalence Rates, and Federal Funding for HIV by State, 2006–2009. Click here for file
